# Detection of Prostate Stem Cell Antigen Expression in Human Prostate Cancer Using Quantum-Dot-Based Technology

**DOI:** 10.3390/s120505461

**Published:** 2012-04-27

**Authors:** Yuan Ruan, Weimin Yu, Fan Cheng, Xiaobin Zhang, Stéphane Larré

**Affiliations:** 1 Department of Urology, Renmin Hospital of Wuhan University, Wuhan 430060, Hubei, China; E-Mails: ruanyuan1982@foxmail.com (Y.R.); ywm.com.cn@163.com (W.Y.); zhangxiaobin1944@yahoo.com.cn (X.Z.); 2 Department of Urology, Angers Teaching Hospital, 4, Rue Larrey, Angers 49000, France; E-Mail: StLarre@chu-angers.fr

**Keywords:** prostate stem cell antigen, prostate cancer, quantum dots, fluorescence

## Abstract

Quantum dots (QDs) are a new class of fluorescent labeling for biological and biomedical applications. In this study, we detected prostate stem cell antigen (PSCA) expression correlated with tumor grade and stage in human prostate cancer by QDs-based immunolabeling and conventional immunohistochemistry (IHC), and evaluated the sensitivity and stability of QDs-based immunolabeling in comparison with IHC. Our data revealed that increasing levels of PSCA expression accompanied advanced tumor grade (QDs labeling, *r* = 0.732, *p* < 0.001; IHC, *r* = 0.683, *p* < 0.001) and stage (QDs labeling, *r* = 0.514, *p* = 0.001; IHC, *r* = 0.432, *p* = 0.005), and the similar tendency was detected by the two methods. In addition, by comparison between the two methods, QDs labeling was consistent with IHC in detecting the expression of PSCA in human prostate tissue correlated with different pathological types (K = 0.845, *p* < 0.001). During the observation time, QDs exhibited superior stability. The intensity of QDs fluorescence remained stable for two weeks (*p* = 0.083) after conjugation to the PSCA protein, and nearly 93% of positive expression with their fluorescence still could be seen after four weeks.

## 1. Introduction

Prostate cancer (PCa) is the most commonly diagnosed cancer and the second leading cause of cancer-related death in American men [1]. Nowadays, it is becoming an increasingly common cancer in China as well. Despite recently great progress in the diagnosis and management of localized disease, these efforts still have been limited by a lack of specific biomarkers of PCa. Therefore, new markers that can accurately discriminate between indolent and aggressive variants of PCa, as well as have the potentialities to be effective therapeutic targets on PCa are imperative.

Prostate stem cell antigen (PSCA), as a cell surface antigen, predominantly expresses prostate specificity. It is a 123 amino acid glycoprotein with 30% identity to stem cell antigen 2 (Sca 2); like Sca-2, PSCA also belongs to a member of the Thy-1/Ly-6 family and is anchored by a glycosylphosphatidylinositol (GPI) linkage. mRNA *in situ* hybridization (ISH) localized PSCA expression in normal prostate to the basal cell epithelium, the putative stem cell compartment of prostatic epithelium, suggesting that PSCA may be a marker of prostate stem/progenitor cells [2].

Semiconductor quantum dots (QDs) are tiny light-emitting particles on the nanometer scale, and are emerging as a new class of fluorescent labels for biology and biomedicine [3]. QDs are generally composed of atoms from elements in groups II-VI or III-V in the periodic table [4,5], and usually consist of a CdSe semiconductor core that is less than 10 nm in diameter, surrounded by an inorganic shell composed of ZnS, which has intrinsic fluorescence emission spectra. This small size results in a quantum confinement effect, which endows nanocrystals with unique optical and electronic properties. In addition, the superior stability attributes to the core-shell with a metal shell structure that passivates the core surface to prevent the intrusion of harsher conditions [6,7] and then the core-shell complex is coated with a polymer to make the particle water soluble, followed by functionalization with streptavidin, for example, to prepare QDs for use in immunochemistry. In comparison with conventional organic dyes and fluorescent proteins, they have novel advantages, such as size- and composition-tunable light emission, superior signal brightness, resistance to photobleaching, narrow and symmetric emission spectra, and broad absorption spectra for simultaneous excitation of multiple fluorescence colors [4,8,9]. These beneficial properties have opened new possibilities for advanced molecular [10,11], cellular [12,13] and *in vivo* imaging [14], as well as for ultrasensitive bioassays and diagnostics [15,16]. In some recent research [17,18], QDs-based immunolabeling was found to display good sensitivity. However, except for sensitivity, it remains to be determined whether QDs fluorescence is stable enough for practical applications in biology and biomedicine. The objective of the present study was to detect prostate stem cell antigen (PSCA) expression correlated with tumor grade and stage in human prostate cancer by QDs-based immunolabeling and conventional immunohistochemistry (IHC), and evaluate the sensitivity and stability of QDs-based immunolabeling in comparison with IHC.

## 2. Experimental Section

### 2.1. Tissue Samples

All tissue specimens were obtained with the permission of the Human Tissue Resources Committee of the Department of Pathology at the Medical College of Wuhan University, Wuhan, China. Eighty formalin-fixed, paraffin embedded human prostate tissues, including 40 cases of prostate cancer (PCa), 20 cases of benign prostatic hyperplasis (BPH), and 20 cases of prostatic intraepithelial neoplasms (PIN), were obtained from 77 patients undergoing transurethral resection of prostate (TURP) or radical prostatectomy at our institution. Blocks were cut into 4-μm sections and mounted on charged slides in the usual fashion and then examined. Section of each PCa sample was graded by an experienced urological pathologist according to the criteria of the Gleason score [19]. Staging was performed based on the 1992 American Joint Committee on Cancer staging system [20]. Gleason scores were categorised into three groups, namely scores of 2–4 (well-differentiation, n = 4), scores of 5–7 (moderate-differentiation, n = 22) and scores of 8–10 (poor-differentiation, n = 14). All specimens in the group with scores 2–4 were stage T1. Five samples in the group with scores 5–7 were T1, and the rest was T2. Nine samples in the group with 8–10 were T2, and the rest was T3.

### 2.2. Quantum-Dots-Based Immunofluorescence Histochemistry

Tissue sections were deparaffinized in xylene and rehydrated in a graded ethanol series. For antibody bindings, slides were first incubated with 2% BSA at 37 °C for 30 min, and then incubation with primary anti-PSCA antibody produced in rabbit (1:100 dilution in TBS, Sigma, St. Louis, MO, USA) overnight at 4 °C, slides were then washed in TBS. Negative control samples were prepared in parallel but the primary antibody was replaced with TBS.

For QDs conjugation, slides were incubated with 2% BSA at 37 °C for 10 min, and then incubated with ZnS-capped CdSe QDs conjugated anti-rabbit IgG probes with an emission wavelength of 605 nm (1:50 dilutions in 2% BSA, Jiayuan Quantum Dot Co. Ltd., China) for 30 min at 37 °C. Following incubation, the slides were vigorously washed with TBS, mounted with neutral glycerol, and stored at 4 °C for observation.

### 2.3. Immunohistochemistry

The same procedures were performed to prepare tissue sections and antigen retrieval. Endogenous enzymes were inactivated by 0.3% hydrogen peroxide at room temperature for 10 min. The tissue samples were then blocked with 5% BSA at 37 °C for 20 min. After 1 h of incubation with primary anti-PSCA antibody produced in rabbit (1:100 diluted in TBS) at 37 °C, slides were washed in TBS. Incubated with biotinconjugated goat anti-rabbit immunoglobulin at 37 °C for 20 min, streptavidin-horseradish peroxidase was then conjugated to goat anti-rabbit immunoglobulin via streptavidin and biotin linking, and antibody localization was performed using the diaminobenzidine reaction. After rinsed with running tap water, counterstaining was performed with hematoxylin. The slides were dehydrated in graded alcohol, cleaned in xylene before mounting in gelatin, and observed under light microscope. TBS was substituted for the primary antibody in negative controls.

### 2.4. Scoring Methods and Image Acquisition

The intensity of PSCA expression evaluated microscopically was graded on a scale of 0 to 3 with 3 being the highest expression observed (0, no staining; 1, mildly intense; 2, moderately intense; 3, severely intense). The staining density was quantified as the percentage of cells staining positive for PSCA with the primary antibody (0, no staining; 1, positive staining in <25% of the sample; 2, positive staining in 25%–50% of the sample; 3, positive staining in >50% of the sample). Intensity score (0–3) was multiplied by the density score (0–3) to give an overall score of 0–9. In this way, we were able to differentiate specimens that may have had focal areas of increased staining from those that had diffuse areas of increased staining. The overall score for each specimen was then categorically assigned to one of the following groups: negative expression (0), 0 score; weak expression (1+), 1–2 scores; moderate expression (2+), 3–6 scores; strong expression (3+), 9 score [21]. All clinical specimen slides were read and quantified by two pathologists in a blinded fashion. There was 95.0% interobserver agreement; any differences were resolved by negotiation.

Fluorescence micrographs were acquired on an Olympus IX 70 fluorescence microscope and imaged by a CCD camera. The QDs were excited by blue light (excitation wavelength of 450–480 nm under U-MWB filters) and present red light under exciting. The immunohistochemistry staining observed under light microscope, and positive cells manifested brown-yellow granular. During the observation period, all labeled slides were stored at 4 °C refrigerator, primarily to prevent drying of tissues.

### 2.5. Statistical Analysis

The consistency of the two methods and comparison of total positive rate in the two methods were analyzed using Cohen's kappa statistic and Chi-square test, respectively. The associations between PSCA expression and Gleason score and clinical stage were calculated using Spearman rank correlation analysis. The intensity and density of PSCA expression in Pca and nonmalignant (BPH and PIN) tissues and variation of different QDs fluorescent scales by time varying were compared using Wilcoxon signed ranks test. All statistical analyses were performed with the SPSS 17.0 statistical package (SPSS Inc.). Significant consistency was considered present when *p* < 0.05.

## 3. Results

### 3.1. PSCA Expression in BPH and PIN

There was no sample with strong expression (3+, composite score 9) in BPH by the two methods. The cases with negative (0, composite score 0), weak (1+, composite score 1–2) and moderate (2+, composite score 3–6) expression were 11 (55%), 3 (15%) and 3 (15%), respectively. In another three (15%) specimens, two (10%) cases were observed with negative expression by IHC, but weak by QDs labeling; and one (5%) case was observed with weak expression by IHC, but moderate by QDs labeling.

In PIN, only one (5%) case was detected with strong expression by the two methods. The cases with negative, weak and moderate expression were seven (35%), three (15%) and six (30%), respectively. In another three (15%) specimens, two (10%) cases were observed with negative expression by IHC, but weak by QDs labeling; and one (5%) case was observed with weak expression by IHC, but moderate by QDs labeling. No statistical difference in PSCA expression levels by the two methods was observed between BPH and PIN (*p* > 0.05).

### 3.2. PSCA Expression in PCa

In general, the strong PSCA expression ratios of PCa were obviously increasing. The cases with negative, weak, moderate and strong expression were one (2.5%), two (5%), 18 (45%) and 16 (40%), respectively. The remaining three (7.5%) specimens with negative to moderate expression by IHC (one negative expression, one weak expression and one moderate expression) had a higher level by QDs labeling (one weak expression, one moderate expression and one strong expression). Overall, the expression levels of PCa by the two methods were significantly higher than nonmalignant (BPH and PIN) specimens (*p* < 0.05, compared with BPH and PIN, respectively).

### 3.3. Correlation of PSCA Expression with Gleason Score and Tumor Stage in PCa

Table 1 shows that, using Spearman rank correlation analysis, the expression levels of PSCA increased significantly with advanced tumor grade in PCa. In addition, a similar tendency was detected by the two methods (QDs labeling, *r* = 0.732, *p* < 0.001; IHC, *r* = 0.683, *p* < 0.001).

With regards to PSCA expression in every stage of PCa, the results are shown in Table 2. The data demonstrated that PSCA expression level and tumor stage in PCa were positively correlated and the two methods manifested a similar tendency as well (QDs labeling, *r* = 0.514, *p* = 0.001; IHC, *r* = 0.432, *p* = 0.005).

### 3.4. Comparison of QDs Labeling and IHC

**Figure 1.**
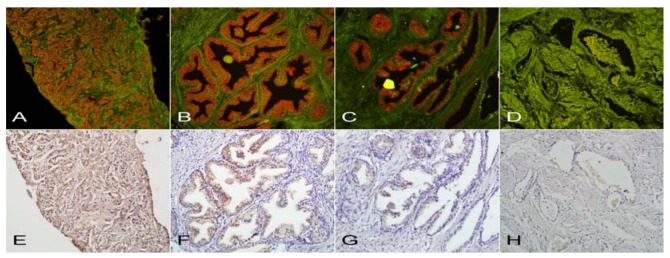
The levels of PSCA expression by QDs labeling and IHC. Under excitation of blue light, (**A**–**C**) show the positive expression of QDs-IHC at 3+, 2+, and 1+ and (**D**) was the negative control. (**E**–**G**) are the positive expression of IHC at 3+, 2+, and 1+ and (**H**) was the negative control. (All magnifications: 200×).

Each level of PSCA expression in prostate tissues by QDs labeling and IHC are shown in Figure 1, and Table 3 displays that QDs labeling was consistent with IHC in detecting the expression of PSCA in human prostate tissue correlated with different pathological types. Overall, the total positive rate (70%, 56/80) of PSCA expression in prostate tissue by IHC was lower than the 76.3% (61/80) measured by QDs labeling, however, there was no statistical difference (*p* = 0.063); Using Cohen's kappa statistic, both methods had statistically similar detection rates for the PSCA expression (K = 0.845, *p* < 0.001).

### 3.5. Optical Stability Test in QDs Labeling

**Figure 2.**
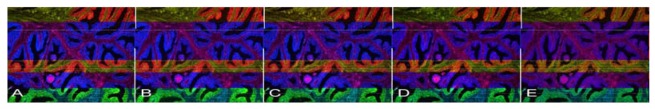
Observation of fluorescence durability on QDs labeling. (**A**–**E**) show the intensity of QDs fluorescence, under excitation of blue light, in the same vision at 0, 7, 14, 21 and 28 days after conjugation to the PSCA protein. (All magnifications: 200×)

**Figure 3.**
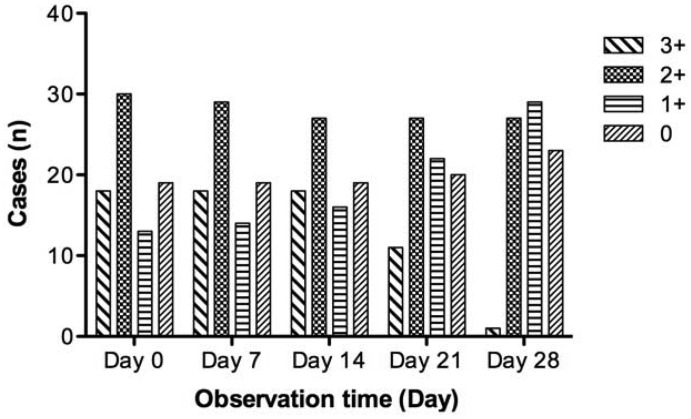
The distribution of different QDs fluorescent scales by time-varying.

Figure 2 shows time-varying images for the QD labeling fluorescence, and the distribution of QD fluorescent intensity for PSCA positive expression variation with time is shown in Figure 3.

On the day of the experiment 61 (76.3%) of the 80 tissue samples showed detectable PSCA expression. In the first two weeks, there was no significant change in the levels of fluorescence intensity. Seven days after the experiment, QD fluorescence remained stable in all but one (1.3%) of the 80 samples (*p* = 0.317). Fourteen days later, only three (3.8%) cases with fluorescence level “2+” faded to “1+” (*p* = 0.083). Twenty-one days after the experiment, the total positive rate experienced a small change, but the variation among each positive expression level was visible (*p* < 0.001). Twenty-eight days later, four (6.6%) of 61 positive samples decayed to 0 and only one sample with strong expression remained (*p* < 0.001).

## 4. Discussion

As a cell surface protein, PSCA marks the earliest phase of hematopoietic development. Its mRNA expresses prostate specificity in normal male tissues and is highly up-regulated in both androgen-dependent and -independent PCa xenografts [21]. PSCA may play an important role in PCa tumorigenesis and progression, and may serve as a target for Pca diagnosis and treatment. In this study, QDs-based immunolabeling and IHC showed that in general there was mainly weak or absent PSCA expression in BPH and PIN tissues, and the variation of PSCA expression levels between them was not marked. However, PSCA was widely expressed in PCa, and the expression levels were significantly higher than BPH and PIN. In addition, the same tendency was displayed by the two methods in that increasing levels of PSCA expression accompanied advanced tumor grade and stage. These data seem to indicate that PSCA is an excellent cell surface marker for human PCa.

In the process of detecting PSCA in prostate tissues, a comparison has been made in the staining technology between QDs-based immunolabeling and IHC. Overall, our data revealed that the two methods had statistically similar detection rates of PSCA expression. According to the results of our consistency analysis, it demonstrated that QD labeling had similar and valuable sensitivity like conventional immunohistochemistry (IHC). Nevertheless, there still remained some differences in the scales of PSCA expression in prostate tissues. On one hand, the total positive rate 70% (56/80) of PSCA expression in prostate tissue by IHC was lower than the 76.3% (61/80) seen by QDs labeling; on the other hand, in some specimens, the results of PSCA expression level by the two methods were inconsistent, and the expression level by QDs labeling was always higher than by IHC. We acknowledge this is a limitation of our study. These differences maybe derive from the stronger signal with a lower background detected by QDs labeling than by IHC. Consequently, in order to eliminate the false positives and prove the accuracy by QDs labeling, a prospective study using a spectral imaging system that can acquire more precise QDs spectra information is needed to verify the superiority of this technology.

Furthermore, with regard to the stability of QDs-based immunolabeling, our results detected that most positive expression with QDs fluorescence could last for four weeks. In the first two weeks, the changes were minor and QD fluorescence was stable in all but three cases. By the beginning of the third week, little change had occurred in the total positive rate, but great variations were found among each positive expression level. Four weeks later, only one sample with strong expression was left, four positive samples had decayed to invisibility, but the fluorescence of nearly 93% of samples with positive expression could still could be seen. The limitation of poor photostability in the traditional organic fluorescent dyes makes them less practical for biology and biomedicine, but these data suggest that QDs fluorescence can remain visible long enough to be potentially valuable for wider applications.

## 5. Conclusions

According to the detection results by QD-based immunolabeling and IHC, PSCA exhibits great advantages for becoming a molecular marker for diagnosis of human PCa. Moreover, superior sensitivity was not only found in QDs-based immunolabeling, but excellent long-term photostability as well. Taking these superiorities together, new opportunities will be provided for fluorescence tracer analysis and *in vivo* imaging, and we anticipate that QD-based technologies will be used widely.

## Figures and Tables

**Table 1.** Associativity analysis of PSCA expression levels and Gleason score in PCa by two methods.

	**Gleason score**				***r***	***p***

	**2–4**	**5–7**	**8–10**	**Total**		
**QDs labeling**						
**0**	1	0	0	1	*r* = 0.732	*p* < 0.001
**1+**	2	1	0	3		
**2+**	0	18	1	19		
**3+**	1	3	13	17		
**Total**	4	22	14	40		

**IHC**						
**0**	2	0	0	2	*r* = 0.683	*p* < 0.001
**1+**	1	2	0	3		
**2+**	0	17	2	19		
**3+**	1	3	12	16		
**Total**	4	22	14	40		

**Table 2.** Associativity analysis of PSCA expression levels and tumor stage in PCa by two methods.

	**Tumor stage**				***r***	***p***

	**T1**	**T2**	**T3**	**Total**		
**QDs labeling**						
**0**	1	0	0	1	*r* = 0.514	*p* = 0.001
**1+**	3	0	0	3		
**2+**	3	16	0	19		
**3+**	2	10	5	17		
**Total**	9	26	5	40		

**IHC**						
**0**	2	0	0	2	*r* = 0.432	*p* = 0.005
**1+**	2	1	0	3		
**2+**	3	15	1	19		
**3+**	2	10	4	16		
**Total**	9	26	5	40		

**Table 3.** Consistency analysis of QDs labeling and IHC technology for detecting the different levels of PSCA expression in various prostate tissues.

**IHC**	**QDs labeling**					**K**	***p***

	**0**	**1+**	**2+**	**3+**	**Total**		
**0**	19	5	0	0	24	K = 0.845	*p* < 0.001
**1+**	0	8	3	0	11		
**2+**	0	0	27	1	28		
**3+**	0	0	0	17	17		
**Total**	19	13	30	18	80		
